# ﻿*Impatienschenmoui* (Balsaminaceae), a new species from southern Yunnan, China

**DOI:** 10.3897/phytokeys.214.94898

**Published:** 2022-11-30

**Authors:** Zheng-wei Wang, Qi Wang, Ru-hua Xu, Yu Zhang, Xiao-chen Li

**Affiliations:** 1 Eastern China Conservation Center for Wild Endangered Plant Resources, Shanghai Chenshan Botanical Garden, Shanghai 201602, China Eastern China Conservation Center for Wild Endangered Plant Resources, Shanghai Chenshan Botanical Garden Shanghai China; 2 Yunnan Yelantang Biological Technology Co., Ltd., Kunming 650114, China Yunnan Yelantang Biological Technology Co., Ltd. Kunming China

**Keywords:** China, *Impatienschenmoui*, morphology, phylogeny, sect. *Uniflorae*

## Abstract

*Impatienschenmoui* (Balsaminaceae), a new species from southern Yunnan, China, was described and illustrated based on morphological and molecular evidence. This new species is morphologically most similar to *Impatiensoblongata* Ruchis. & Niet, but can be distinguished by 7–9 pairs of leaf veins, glabrous perianth, obovate upper petal, and capsule with trichome.

## ﻿Introduction

The family Balsaminaceae contains two genera, the monotypic *Hydrocera*Blume (1825:241) and *Impatiens*Linnaeus (1753: 937) (APG Website, http://www.mobot.org/MOBOT/research/APweb/) *Impatiens* L. is a large genus of over 1000 species (Grey-Wilson 1980; Fischer 2004), mainly distributed in tropical and subtropical regions, with tropical Africa, Madagascar, southern India and Sri Lanka, eastern Himalayas (including SW China) and Southeast Asia as its five diversity centers (Song et al. 2003; Yuan et al. 2004; Yu et al. 2015). More than 270 species are currently known from China (Yu 2012), in which more than 200 species occurred in SW China (Chen et al. 2007), mainly distributed in Yunnan, Sichuan, Guangxi, Guizhou, and Xizang. *Impatiens* was divided into two subgenera, subgen. Clavicarpa S.X. Yu ex S.X. Yu & Wei Wang and subgen. Impatiens L. according to the latest phylogenetic studies. The latter was further subdivided into seven sections (sect. Fasciculatae, sect. Impatiens, sect. Racemosae, sect. Scorpioidae, sect. Semeiocardium, sect. Tuberosae, and sect. Uniflorae) (Yu et al. 2015). Several new species of sect. Uniflorae have been described from India, Myanmar, Cambodia, Vietnam, and China. (e.g. Cho et al. 2017; Yang et al. 2017; Ruchisansakun et al. 2018; Kim et al. 2019; Zhang et al. 2020) in recent years.

In September 2019, during fieldwork in Mengla County, Yunnan, an unfamiliar *Impatiens* species was collected and transplanted to Shanghai Chenshan Botanical Garden. The flower blossomed in December 2020, indicating its unusual identity which may be new to science. In November 2021, we made a botanical trip back to Mengla County to collect flowers and fruit specimens. After careful comparison of relevant species from the adjacent area, we finally concluded that these specimens represent a species new to science, and described it here.

## ﻿Methods

### ﻿Morphology study

Morphological characters of the new species and related ones were compared based on living plants and herbarium specimens, including the digital resource of type specimens from JSTOR Global Plants (https://plants.jstor.org/). Herbarium specimens were examined in Chenshan Botanical Herbarium (CSH, index herbarium, http://sweetgum.nybg.org/science/ih/herbarium-list/?NamOrganisationAcronym=CSH), original protologues and relevant literature were also investigated.

### ﻿Datasets preparation

To resolve the phylogenetic position of the putative new species, two molecular markers ITS (ITS1–5.8S-ITS2) and *atpB-rbcL* were used in this study. Leaf material of the putative new species was collected from the field and stored with silica. Total genomic DNA was extracted with the modified CTAB method (Doyle and Doyle 1987) for library construction at Benagen (https://www.benagen.com). Paired-end sequencing of the whole sequences from both ends of 150 bp fragments was performed on the DNBSEQ T7, and about 2 Gb clean data were produced. The plastome and nrDNA were de novo assembled using the GetOrganelle pipeline 1.7.6.1 (Jin et al. 2020). Sequences of *atpB-rbcL* were extracted from the plastome annotated in Geneious Prime 2021.2.2 (https://www.geneious.com) with comparison to the published plastome of *Impatiensbalsamina* L. (GenBank accession: MW411292) as reference. Sequences of ITS1–5.8s-ITS2 were extracted with ITSx 1.1.3 (Bengtsson-Palme et al. 2013). The ITS dataset and the *atpB-rbcL* dataset were respectively aligned using MAFFT v7.450 by default setting. (Katoh and Standley 2013) and concatenated for phylogenetic analysis (Chen et al. 2020). Species sampling was based on previous studies (Yu et al. 2015; Ruchisansakun et al. 2018). All the sequence GenBank accession numbers were listed in Appendix 1.

### ﻿Phylogenetic analysis

Maximum Likelihood estimation (ML) and Bayesian inference analysis (BI) were performed on Phylosuite v1.2.2 (Zhang et al. 2020). For ML, GTR+F+R4 was selected as the best fit model for the ITS dataset, and GTR+F+R5 was selected as the best fit model for the *atpB-rbcL* dataset according to AICc by Modelfinder (Kalyaanamoorthy et al. 2017). Maximum likelihood was estimated using IQ-TREE (Nguyen et al. 2015) under the Edge-linked partition model for 2000 ultrafast (Minh et al. 2013) bootstraps. For BI, GTR+I+G was selected as the best fit model for both datasets according to AICc by PartitionFinder2 (Lanfear et al. 2017). Bayesian Inference phylogeny analysis was inferred using MrBayes 3.2.6 (Ronquist et al. 2012) under the partition model (2 parallel runs, 10,000,000 generations), in which the initial 25000 sampled data were discarded as burn-in. Tree files were visualized and annotated in Figtree v1.4.4 (http://tree.bio.ed.ac.uk/software/figtree/). Bootstrap (BS) and Posterior Probability (PP) values were used as an estimate of nodal robustness.

## ﻿Result

**Figure 1.**
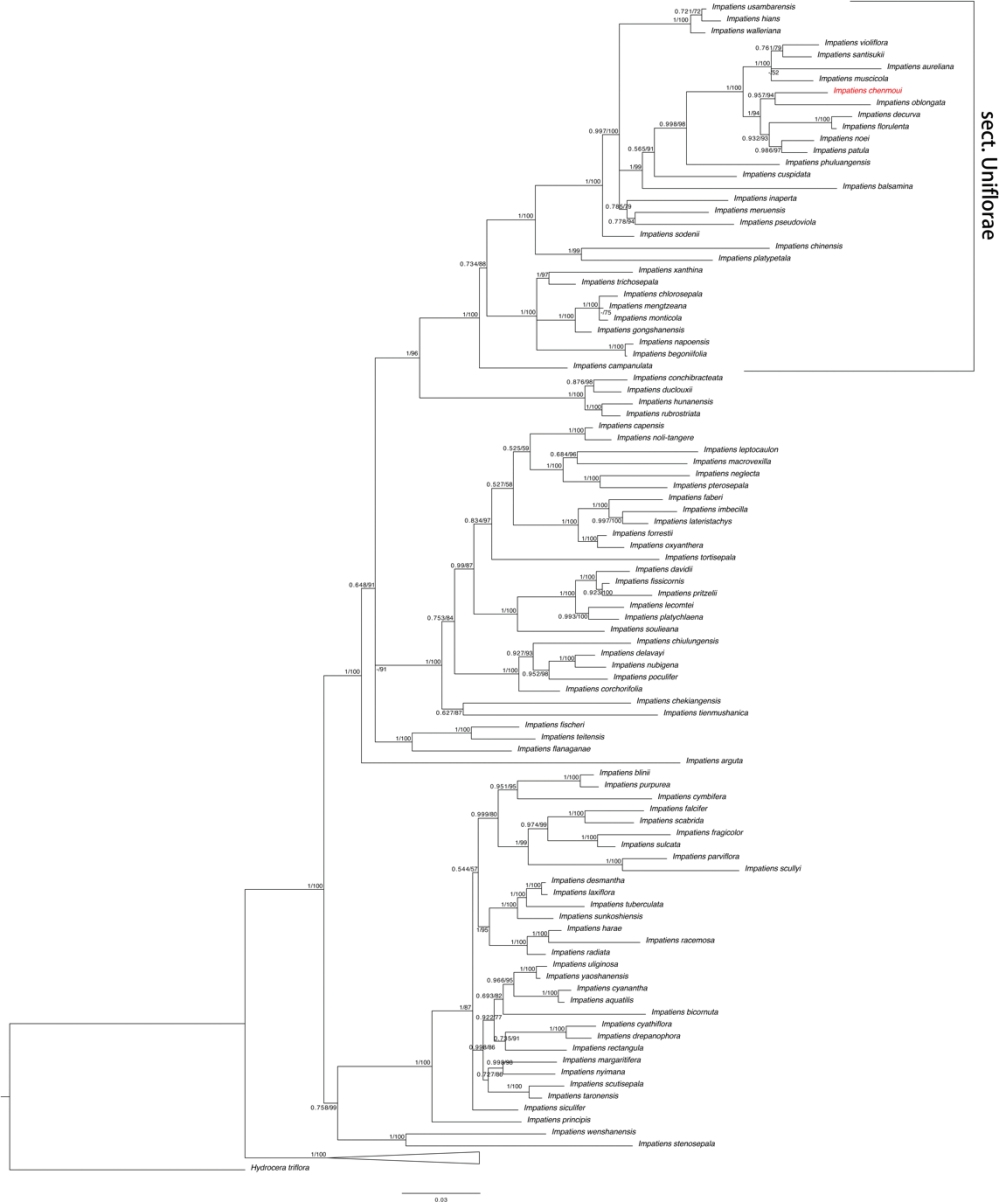
Phylogenetic tree based on combined datasets of the nuclear ITS and plastid *atpB–rbcL* DNA sequences. The topological structure comes from Bayesian inference. Numbers near nodes are PP/BS, a dash ‘–’ indicates nodes not supported, subgen. Clavicarpa was collapsed.

The combined dataset was 1934bp in total, compromising 107 accessions/107 species, with *Hydroceratriflora* (L.) Wight. et Arn. selected as outgroup. Phylogenetic reconstruction of BI and ML produced similar topological structures (Fig. 1). The putative new species (marked in red) was resolved in the subgen. Impatienssect.Uniflorae, forming a sister relationship with Myanmar species *I.oblongata* Ruchis. & Niet (PP = 0.957, BS = 94). Based on the morphological characters and phylogenetic result, we recognized this *Impatiens* species as a new species and described it here as *Impatienschenmoui* Zheng W. Wang, Xiao C. Li & Qi Wang, sp. nov.

### ﻿Taxonomic treatment

#### ﻿ Impatiens chenmoui

Taxon classificationPlantaeEricalesBalsaminaceae

Zheng W. Wang, Xiao C. Li & Q.Wang ter sp. nov.

9CCC0B61-277D-5E48-81A6-C67E78A1F937

urn:lsid:ipni.org:names:77309066-1

**Figure 2.**
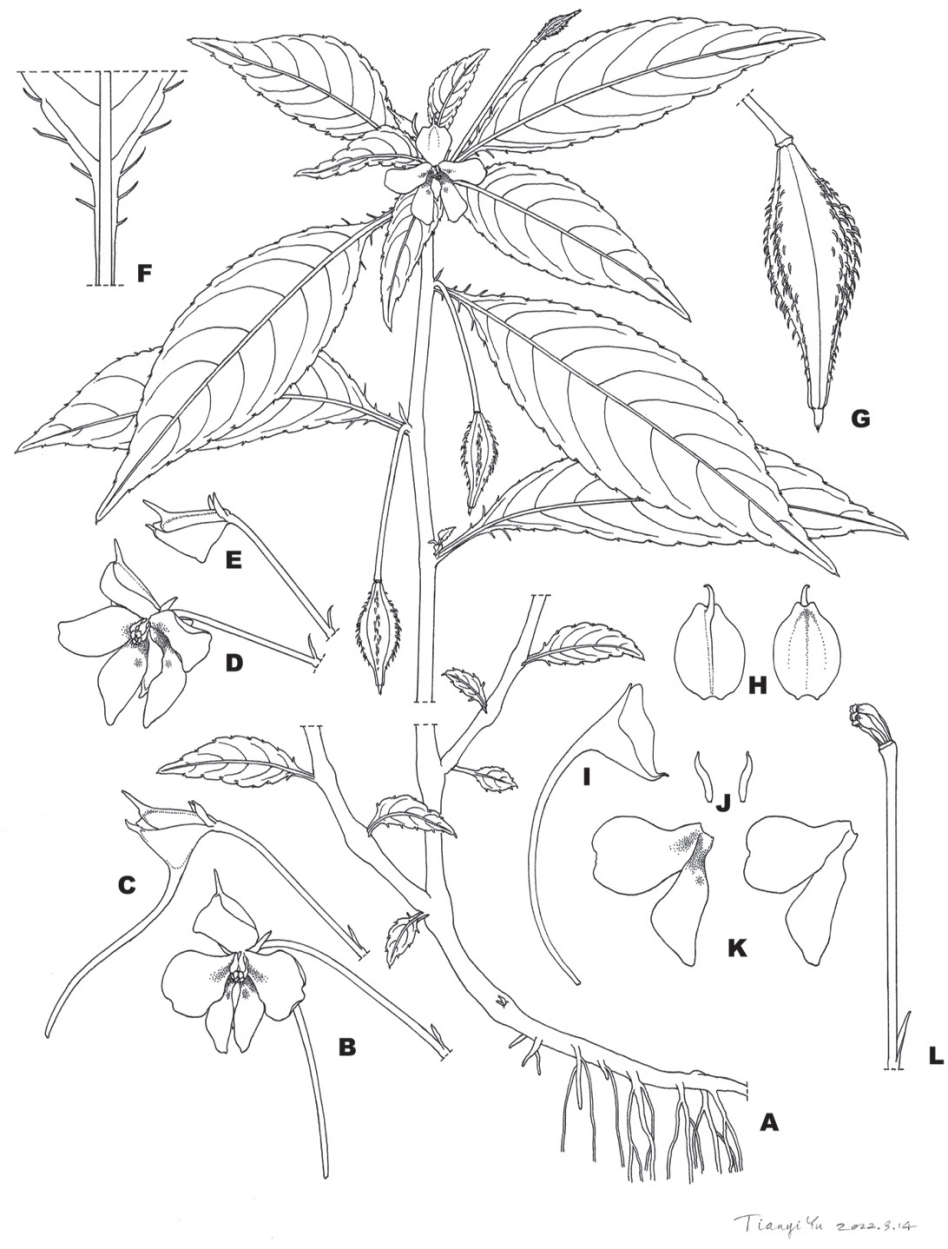
*Impatienschenmoui* sp. nov. **A** habit **B, C** flower with long spur **D, E** flower with spur nearly absent **F** leaf base **G** capsule **H** dorsal petal **I** spur **J** lateral sepals **K** united lateral petals **L** ovary surrounded by stamens.

**Figure 3.**
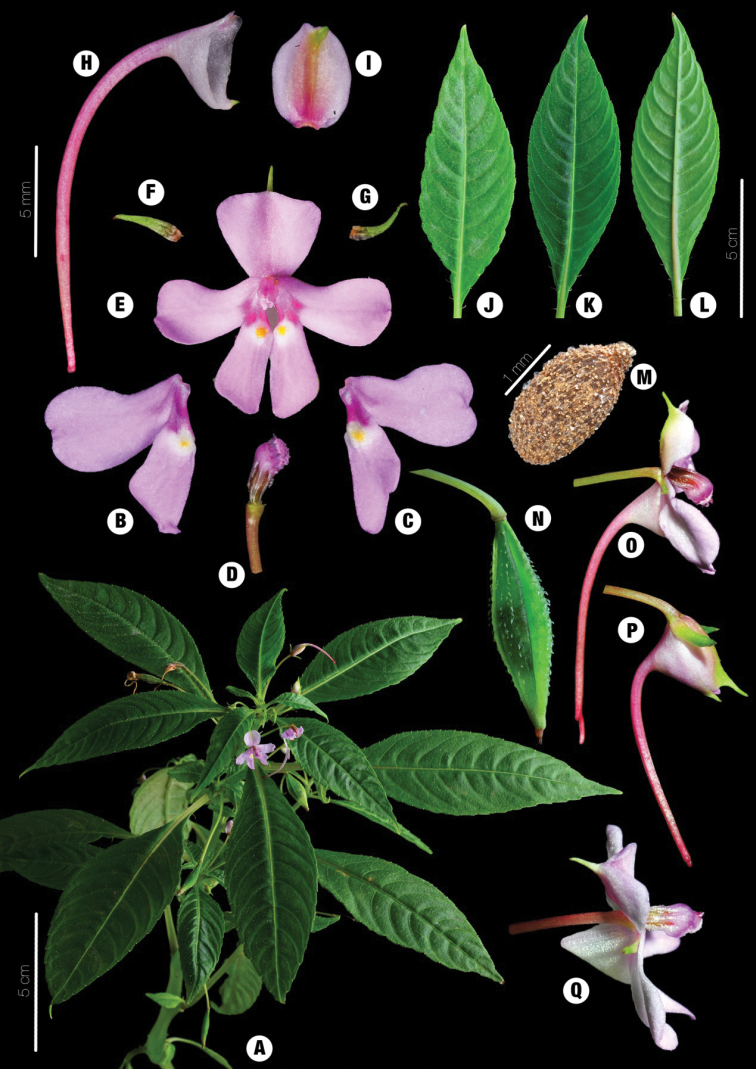
*Impatienschenmoui* sp. nov. **A** habit **B, C** united lateral petals **D** ﻿ovary surrounded by stamens **E** flower front view **F, G** lateral sepals **H** spur **I** dorsal petal **J–L** leaves **M** seed **N** capsule **O, P** long-spurred flower side view **Q** non-spurred flower side view.

Figs 2 , 3 , Appendix 2

##### Type.

China. Yunnan province, Mengla county (勐腊县) Xiangming Yi nationality township (象明彝族自治乡) Kongming Mountain (孔明山) alt.1639m, 22°8'9.73"N, 101°8'48.86"E, 23 November 2021, *Zhengwei Wang and Xiaochen Li*, *WZW04250* (Holotype: CSH0189505, CSH!; isotypes: CSH0192380, ZJFC!; CSH0189507, HZU!; CSH0189506, JJF!).

##### Diagnosis.

*Impatienschenmoui* is most similar to *I.oblongata* Ruchis. & Niet, but is distinguished by the glabrous dorsal petal, pedicel, and bracts, longer pedicel and spur, and fewer lateral sepals (Table 1).

**Table 1.** Comparison of key features of *I.chenmoui* and *I.oblongata*.

Taxonomic traits	*I.chenmoui*	*I.oblongata*
Dorsal petal	Glabrous	midrib and tip pilose
Pedicel	25–27 mm long, green, glabrous.	18–20 mm long, pink, pilose.
Ovary hair	Trichome	Pilose
Spur	14–17 mm long, glabrous, rarely absent.	8–12 mm long, pilose.
Bracts	Glabrous	Pilose
Lateral sepals	2, inversely coiled, glabrous	2–4, upper pair pilose; lower pair glabrous

##### Description.

Herb annual. Stem erect, fleshy, glabrous, 12–35 cm tall. Leaves alternate, petioles 1–5 cm, leaf blade 9.5–2.5×1.6–3.5 cm, narrowly elliptic or oblong-lanceolate, apex acuminate or long acuminate, base cuneate, margin roughly crenate; adaxially dark green, pilose along veins, abaxially gray-green, glabrous, lateral veins 7–9 pairs. Inflorescences in upper leaf axils, 1–flowered. Pedicels green, glabrous, 2.5 –2.7 cm long. Bracts linear, persistent, 2–3 mm long. Flowers solitary, axillary, pink, or lavender, with pair of darker pink and yellow dots at the base. Lateral sepals 2, inversely coiled, glabrous, green, 2 mm long. Lower sepal funnelform, 3–4×2–3mm long, 2–3mm in depth, eaves navicular, base gradually constricted into a spur, variable, usually1.4–1.7 cm long, rarely absent, mouth oblique, ca. 5mm wide, with ca. 2 mm long narrowly triangular tip. Dorsal petal circular, pink or mauve, 5–6×4–5mm, apex acuminate, glabrous, ca. 2mm long. United lateral petals sessile, 2–lobed, 6–8mm long. Upper petal large, obovate, 6–7×2–5mm, apex often concave. Lower petal small, axe-shaped, 7–8×1–3mm, apex rounded, without auriculus at back. Stamens 5, filaments linear, subulate, pale pink, ca. 2–3mm long, distally enlarged, anthers obtuse. Ovary fusiform, 5–carpellate, purple, 2–3 mm long, dorsal suture ridges with trichome. Capsule short fusiform, 12–18mm long, 4–5 mm in diam, with trichome along ridges. Seeds obovoid, brown, ca. 2 mm long, slightly compressed, pubescent with spirally sculptured hairs.

##### Phenology.

Flowering and fruiting from October to December.

##### Distribution and ecology.

This new species was found under evergreen broad-leaved forest at an elevation of 1500–1700 m on the limestone mountain ridge, and was currently known as only one population in Mengla County, Yunnan, China. This distribution area is very close to the border with Myanmar and Laos. We assume that this species should be also distributed in Myanmar and Laos due to their similar habitat.

##### Etymology.

The specific epithet “Chenmoui” was dedicated to the famous Chinese collector and botanist, Chen Mou (陈谋) (1903–1935) who was one of the founders of the first botanical garden cataloged by the Classification System of Plants in China, and died during the collection trip through southern Yunnan, China. The Chinese name was given as “陈谋凤仙花”.

##### Conservation status.

This species is currently known only from one population in the type locality. The population is located in the tourist area of Kongming Mountain, where it could be easily disturbed by human activities, such as road construction and illegal mining. The IUCN status proposed is Vulnerable(VU) based on IUCN (2022) guidelines.

##### Additional specimens examined

**(Paratype).** China,Yunnan province, Mengla county, Xiangming Yi nationality township, Kongming Mountain. 24 Oct. 2019, *Ruhua Xu and Yu Zhang*, *XRH001* (CSH!).

##### Note.

New species of sect. Uniflorae discovered from Southeast Asia in recent years were mostly found distributed on mountain summits in an evergreen forest, which indicated that the stone mountain in this area was likely to be one of the speciation centers of this section. Impatiens species exhibited interspecific and even intraspecific variation in spur length, at least from our observation of the same population of *I.davidii* Franchet, *I.platysepala* Y. L. Chen, and *I.chenmoui*, which may be considered as retaining of a bimodal pollinated system of bee and lepidopteran (Ruchisansakun et al. 2016). Floristic survey and pollination ecology study in these regions’ *Impatiens* species is still insufficient, and more fieldwork is urgently needed.

## Supplementary Material

XML Treatment for Impatiens chenmoui

## Figures and Tables

**Figure A1.**
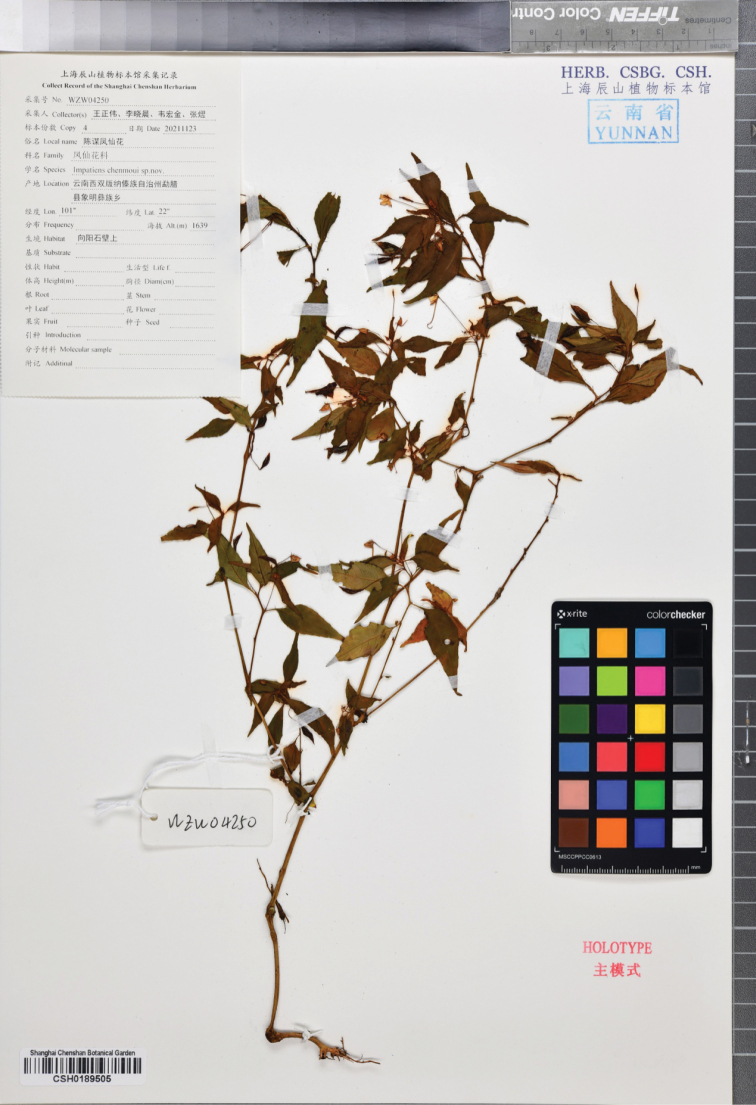
Holotype of *Impatienschenmoui* sp.nov.

## ﻿Appendix 1

**Table A1.** Species and sequences sampling list with Genbank accession number.

Species	ITS	*atpB–rbcL*
*Hydroceratriflora*	AY348853	DQ147895
*Impatiensapalophylla*	KP776061	KP776011
*Impatiensaquatilis*	AY348745	DQ147811
*Impatiensarguta*	AY348746	DQ147812
*Impatiensaureliana*	AY348747	DQ147814
*Impatiensbalansae*	KP776062	KP776012
*Impatiensbalsamina*	AY348749	DQ147816
*Impatiensbegoniifolia*	AY348752	DQ147819
*Impatiensbicornuta*	AY348754	DQ147821
*Impatiensblinii*	KP776063	KP776013
*Impatienscampanulata*	AY348758	DQ147822
*Impatienscapensis*	AY348759	DQ147823
*Impatienschekiangensis*	KP776064	KP776014
*Impatienschenmoui*	OP035808	OP095354
*Impatienschinensis*	AY348761	DQ147825
*Impatienschishuiensis*	KP776065	KP776015
*Impatienschiulungensis*	KP776066	KP776016
*Impatienschlorosepala*	KP776067	KP776017
*Impatiensclavigera*	KP776068	KP776018
*Impatiensconchibracteata*	AY348765	DQ147829
*Impatienscorchorifolia*	AY348767	DQ147831
*Impatienscuspidata*	AY348769	DQ147832
*Impatienscyanantha*	AY348770	DQ147833
*Impatienscyathiflora*	AY348771	DQ147834
*Impatienscymbifera*	KP776069	KP776019
*Impatiensdavidii*	KP776070	KP776020
*Impatiensdecurva*	MF979085	MF979082
*Impatiensdelavayi*	AY348773	DQ147836
*Impatiensdesmantha*	AY348774	DQ147837
*Impatiensdrepanophora*	AY348776	DQ147838
*Impatiensduclouxii*	KP776071	KP776021
*Impatiensfaberi*	AY348778	DQ147841
*Impatiensfalcifer*	KP776072	KP776022
*Impatiensfischeri*	AY348781	DQ147843
*Impatiensfissicornis*	AY348782	DQ147844
*Impatiensflanaganae*	AY348783	DQ147846
*Impatiensflorulenta*	MF979087	MF979084
*Impatiensforrestii*	AY348784	DQ147847
*Impatiensfragicolor*	KP776073	KP776023
*Impatiensgongshanensis*	KP776074	KP776024
*Impatiensharae*	KP776075	KP776025
*Impatienshians*	AY348791	DQ147849
*Impatienshongkongensis*	KP776076	KP776027
*Impatienshunanensis*	KP776077	KP776028
*Impatiensimbecilla*	AY348796	DQ147851
*Impatiensinaperta*	AY348797	DQ147852
*Impatienslateristachys*	KP776078	KP776030
*Impatienslaxiflora*	KP776079	KP776031
*Impatienslecomtei*	AY348802	DQ147855
*Impatiensleptocaulon*	KP776080	KP776032
*Impatiensmacrovexilla*	KP776082	KP776034
*Impatiensmalipoensis*	KP776083	KP776035
*Impatiensmargaritifera*	KP776084	KP776036
*Impatiensmengtzeana*	AY348806	DQ147858
*Impatiensmeruensis*	AY348807	DQ147859
*Impatiensmonticola*	AY348810	DQ147860
*Impatiensmuscicola*	KC905500	KC905547
*Impatiensnapoensis*	AY348811	DQ147861
*Impatiensneglecta*	KP776087	KP776038
*Impatiensnoei*	KC905504	KC905548
*Impatiens noli–tangere*	KP776088	KP776039
*Impatiensnubigena*	KP776089	KP776040
*Impatiensnyimana*	KP776090	KP776041
*Impatiensoblongata*	MF979086	MF979083
*Impatiensomeiana*	KP776092	DQ147864
*Impatiensoxyanthera*	AY348814	DQ147865
*Impatiensparviflora*	AY348816	DQ147866
*Impatienspatula*	KC905509	KC905549
*Impatiensphuluangensis*	KC905517	KC905554
*Impatiensplatychlaena*	AY348818	DQ147867
*Impatiensplatypetala*	AY348819	DQ147868
*Impatienspoculifer*	AY348820	DQ147870
*Impatiensprincipis*	KP776096	KP776026
*Impatienspritzelii*	AY348821	KP776045
*Impatienspseudoviola*	AY348822	DQ147871
*Impatienspterosepala*	KP776097	KP776046
*Impatienspurpurea*	AY348823	DQ147872
*Impatiensracemosa*	KP776098	DQ147873
*Impatiensradiata*	AY348824	KP776047
*Impatiensrectangula*	AY348825	DQ147874
*Impatiensrubrostriata*	AY348828	DQ147876
*Impatienssantisukii*	KC905528	KC905550
*Impatiensscabrida*	KP776099	DQ147877
*Impatiensscullyi*	KP776100	KP776048
*Impatiensscutisepala*	AY348830	DQ147878
*Impatienssiculifer*	KP776101	KP776049
*Impatienssodenii*	AY348832	DQ147879
*Impatienssoulieana*	AY348833	DQ147880
*Impatiensspathulata*	KP776102	KP776050
*Impatiensstenosepala*	AY348835	DQ147881
*Impatienssulcata*	KP776103	KP776051
*Impatienssunkoshiensis*	KP776104	KP776052
*Impatienstaronensis*	AY348838	DQ147882
*Impatiensteitensis*	AY348840	DQ147883
*Impatienstienmushanica*	KP776105	KP776053
*Impatienstortisepala*	KP776106	KP776054
*Impatienstrichosepala*	AY348843	DQ147885
*Impatienstuberculata*	KP776107	KP776055
*Impatienstubulosa*	KP776108	KP776056
*Impatiensuliginosa*	AY348845	DQ147887
*Impatiensusambarensis*	AY348847	DQ147890
*Impatiensvioliflora*	KC905541	KC905551
*Impatienswalleriana*	AY348849	DQ147892
*Impatienswenshanensis*	KP776110	KP776057
*Impatienswilsonii*	KP776111	KP776058
*Impatiensxanthina*	AY348850	DQ147893
*Impatiensyaoshanensis*	KP776112	KP776059
